# Review of Atrioventricular Node Ablation Combined with Permanent His-Purkinje Conduction System Pacing in Patients with Atrial Fibrillation with Heart Failure

**DOI:** 10.31083/j.rcm2509312

**Published:** 2024-09-05

**Authors:** Lina Wang, Chen Tan, Jingshu Lei, CHONGYOU LEE

**Affiliations:** ^1^Department of Cardiology, Beijing Key Laboratory of Early Prediction and Intervention of Acute Myocardial Infarction, Peking University People's Hospital, 100035 Beijing, China; ^2^Department of Cardiology, Hebei Yanda Hospital, 065201 Langfang, Hebei, China

**Keywords:** His-Purkinje conduction system pacing, atrioventricular node ablation, heart failure, atrial fibrillation, His bundle pacing, left bundle branch pacing

## Abstract

With the advancement of pacing technologies, His-Purkinje conduction system pacing (HPCSP) has been increasingly recognized as superior to conventional right ventricular pacing (RVP) and biventricular pacing (BVP). This method is characterized by a series of strategies that either strengthen the native cardiac conduction system or fully preserve physical atrioventricular activation, ensuring optimal clinical outcomes. Treatment with HPCSP is divided into two pacing categories, His bundle pacing (HBP) and left bundle branch pacing (LBBP), and when combined with atrioventricular node ablation (AVNA), can significantly improve left ventricular (LV) function. It effectively prevents tachycardia and regulates ventricular rates, demonstrating its efficacy and safety across different QRS wave complex durations. Therefore, HPCSP combined with AVNA can alleviate symptoms and improve the quality of life in patients with persistent atrial fibrillation (AF) who are unresponsive to multiple radiofrequency ablation, particularly those with concomitant heart failure (HF) who are at risk of further deterioration. As a result, this “pace and ablate” strategy could become a first-line treatment for refractory AF. As a pacing modality, HBP faces challenges in achieving precise localization and tends to increase the pacing threshold. Thus, LBBP has emerged as a novel approach within HPCSP, offering lower thresholds, higher sensing amplitudes, and improved success rates, potentially making it a preferable alternative to HBP. Future large-scale, prospective, and randomized controlled studies are needed to evaluate patient selection and implantation technology, aiming to clarify the differential clinical outcomes between pacing modalities.

## 1. Introduction

**Fig. 1.**
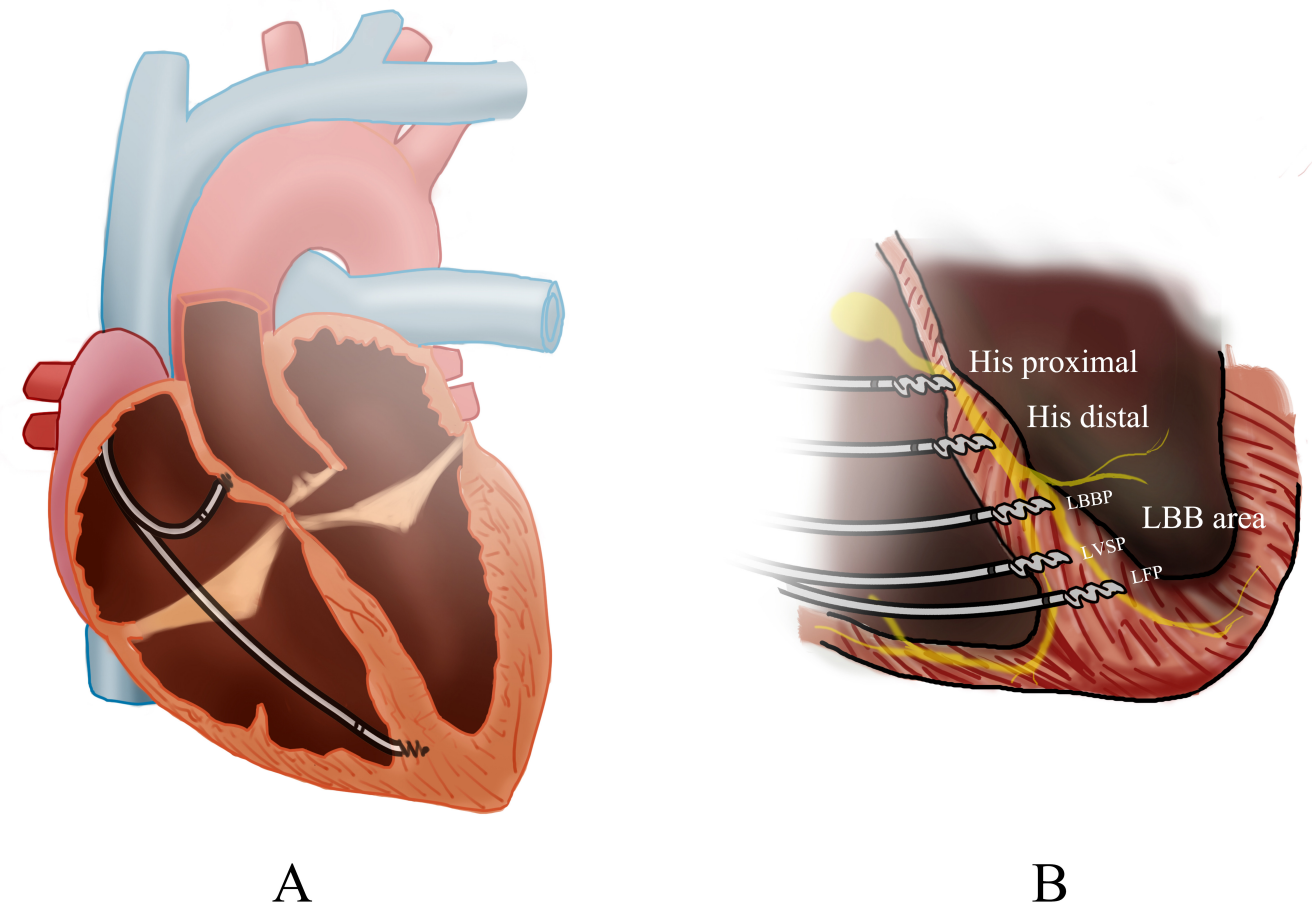
**Overview of cardiac pacing modalities**. (A) The biventricular pacing (BiVP) general view. (B) The pacing of different His-Purkinje Conduction System Pacing (HPCSP) region. LBBP, left bunch bundle pacing; LVSP, left ventricular septal pacing; LFP, left fascicular pacing; LBB, Left bundle branch.

Cardiac pacing effectively alleviates symptoms in patients with atrial fibrillation (AF) and congestive heart failure (HF) following radio-frequency ablation [1]. As physiological pacing has evolved, many cardiac pacing modalities have been developed, including right ventricular pacing (RVP), biventricular pacing (BVP), and His-Purkinje conduction system pacing (HPCSP) (Fig. 1) [2]. Multiple studies have shown that RVP can lead to increases in the QRS duration, both interventricular and intraventricular electrical desynchrony, and left ventricular (LV) function deterioration [3, 4, 5]. Conversely, BVP, when combined with atrioventricular node ablation (AVNA) has shown improved outcomes [6]. The 2021 European Society of Cardiology (ESC) Guidelines recommended cardiac resynchronization therapy (CRT) for patients with permanent AF and HF with a left ventricular ejection fraction (LVEF) ≤35% and a QRS ≥130 ms [6]. However, the benefits of CRT may be less significant in patients with symptomatic AF, moderately reduced ejection fraction (EF), and a narrow QRS [7, 8]. Moreover, patients with right bundle branch block (RBBB) are at a higher risk of not responding or even experiencing a negative response to traditional BVP [9, 10].

Pacing through HPCSP is considered superior to conventional RVP and BVP, especially in the form of His bundle pacing (HBP), which is thought to most closely mimic physiological ventricular contraction and thus prevent long-term ventricular dysfunction [11]. In 2000, Deshmukh *et al*. [12] demonstrated that HBP could improve LV dimensions and cardiac function in a small group of patients with AF and dilated cardiomyopathy. Subsequent studies have highlighted the advantages of HBP in preserving electrical synchrony and LV function when compared to conventional RVP [11, 13, 14]. Guidelines now give HBP a class IIa recommendation for patients with a reduced LVEF between 35% and 50% [15, 16]. However, the HBP threshold could increase by 0.5–1.5 V following AVNA [17]. Additionally, the physiological reliance on HPCSP leads to pacer-dependence, which differs from the completely native intrinsic conduction system [18]. Therefore, we conducted this review to evaluate the efficacy of permanent HPCSP in patients undergoing AVNA for AF with HF.

## 2. HPCSP and AV Node Ablation (AVNA)

The HPCSP benefits from strategies that strengthen either the native cardiac conduction system or preserve physical atrioventricular activation, improving clinical outcomes [19, 20]. Based on pacing, HPCSP can be divided into two main categories: HBP and left bundle branch pacing (LBBP) [21]. Both modalities can improve electromechanical synchronization, leading to more synchronized ventricular contractions [21]. This makes them viable alternatives for achieving CRT, regardless of the QRS duration. In contrast, non-response to CRT still remains high, ranging between 30% and 40% [22, 23], and only patients with a typical LBBB QRS pattern exhibit improved responses to CRT [24]. Meanwhile, an increasing number of clinical studies have exhibited the feasibility, safety, and clinical benefits of HPCSP in comparison with standard RVP [11, 25]. Although HPCSP after AVNA can alleviate symptoms and improve the quality of life in patients with persistent AF who are refractory to multiple radiofrequency ablations, this pacing modality has not obtained widespread adoption in clinical practice owing to various challenges.

The HBP is stretches from the compact atrioventricular node to the membranous interventricular septum [26]. His bundle capture can be either selective and non-selective [14, 26]. Selective capture targets only the His bundle, resulting in a paced rhythm identical to the intrinsic QRS, with an isoelectric interval between the pacing stimulus and the onset of ventricular activation. Non-selective capture occurs when both the His bundle and the surrounding basal septal myocardium are engaged, producing a pseudo-delta morphology on the electrocardiogram (ECG) with a slightly broader QRS duration [14, 26]. Observational studies have not shown significant differences in clinical benefits between the two methods [27]. Similarly, limited studies have shown no significant differences in clinical endpoints between patients undergoing selective and non-selective HBP [27]. Further mechanistic studies had also confirmed that the left ventricular activation time and left ventricular synchronization are similar for both [28].

Following its initial introduction by Huang *et al*. [29], LBBP has emerged as a novel approach for HPCSP with the capability to overcome sites of conduction block while maintaining low and stable capture thresholds. Initially, LBBP can capture either the left branch bundle (LBB) alone, the LBB along with nearby myocardium, or just the adjacent myocardium before extending to the LBB, ultimately activating the complete left ventricle [30]. Importantly, the pacing lead for LBB can positioned distal to any vulnerable or pathological areas, minimizing potential damage to the conduction system during implantation [31]. Therefore, the LBB covers a broader area of the left ventricular septum compared to the HBP, providing a more extensive region for effective pacing [31].

Generally, AVNA is reserved for refractory AF cases where all other therapeutic strategies have failed or been ruled out, with a permanent pacemaker being implanted following the procedure [15]. The primary benefit of this strategy is the prevention of tachycardia and maintenance of regular and controlled ventricular rates [32]. Consequently, HPCSP represents an ideal physiological pacing option to prevent ventricular desynchrony in patients undergoing AVNA. The AVNA procedure is often carried out concurrently with HPCSP or performed 4–6 weeks following the pacing implantation [26]. This delay allows the ventricular lead to stabilize, ensuring regular ventricular pacing thresholds prior to performing AVNA [26]. According to the 2019 ESC Guidelines, AVNA followed by pacing—whether biventricular or HBP—is recommended if tachycardia that contributes to tachycardia-induced cardiomyopathy (TCM) cannot be ablated or controlled by medication [33]. This strategy has a class I recommendation and a level of evidence C [33].

## 3. AVNA Combined with HPCSP Has Advantages in Patients with AF Accompanied HF

Suitable for patients with both wide and narrow QRS, HPCSP is effective for treating patients with either heart failure with reduced ejection fraction (HFrEF) or heart failure with preserved ejection fraction (HFpEF) [34]. When combined with HPCSP, AVNA was found to be effective and safe in most patients with persistent AF and HF [35], simplifying drug treatment in patients with HF. Furthermore, a comprehensive overview of studies highlighting the efficacy of combining AVNA with HPCSP in patients with AF and HF is presented in Table 1 (Ref. [12, 18, 35, 36, 37, 38, 39, 40, 41, 42]). This table summarizes the main studies, including population characteristics, study design, and outcomes, offering a consolidated view of the research advancements in this area.

**Table 1.** **Summary of Key Studies on AVNA Combined with HPCSP in Patients with AF with HF**.

Study	Year	Population	Design	Indication	Age (years)	Number	Follow-up time (months)	Implant success (%)
Deshmukh *et al*. [12]	2000	Chronic AF, dilated cardiomyopathy, and normal activation	Prospective observational, single-arm	DHBP+AVNA	69 ± 10	18	23.4 ± 8.3	14 (66)
Deshmukh *et al*. [39]	2004	Cardiomyopathy, persistent AF	Single-arm	DHBP+AVNA	70 ± 8	54	42 (72.2)	29 (53.7)
Occhetta *et al*. [38]	2006	Chronic AF	Randomised, crossover, blinded, single-arm	AVNA+HBP	71.4 ± 5.6	36	12	18 (50)
Vijayaraman *et al*. [18]	2017	Persistent and Paroxysmal AF and difficulty in rate control	Retrospective observational, single-arm	AVNA+HBP	74 ± 11	42	19 ± 14	40 (95)
Huang *et al* [37]	2017	Symptomatic long-lasting persistent or permanent AF with narrow QRS and HF	Prospective observational, single-arm	AVNA+HBP	72.8 ± 8.3	52	21.1 ± 9.3	42 (80.8)
Wang *et al*. [35]	2019	Persistent AF and heart failure	Retrospective, case-control	HBP+AVNA	67.60 ± 10.85	54	12	52 (94.5)
Su *et al*. [36]	2020	Persistent AF with symptomatic HF and narrow QRS	Single-arm	AVNA+HBP+LBBP	70.3 ± 10.0	94	36	81 (86.2)
Ye *et al*. [40]	2023	Rapid AF	Single-arm	HBP +LBBP+AVNA	NR	16	6	10 (81.2)
Moriña-Vázquez *et al*. [41]	2021	AF	Single-arm	HBP+AVNA	77 (70–81)	39	10.5	36 (92.3)
Žižek *et al*. [42]	2022	AF, narrow QRS	Retrospective, case-control	HBP+AVNA	68.5 (6.8)	16	6	12 (75)

AVNA, atrioventricular node ablation; HPCSP, His-Purkinje conduction system pacing; AF, atrial fibrillation; HBP, His bundle pacing; LBBP, left bundle-branch pacing; NR, not reported; HF, heart failure; DHBP, direct His-bundle pacing.

Delving into specifics, Su *et al*. [36] found the combination of HBP with AVNA effectively reduced the New York heart association (NYHA) functional class, cardiothoracic ratio (CTR), left ventricular end-systolic volume (LVESV), and the need for diuretics or digoxin, while increasing LVEF in patients both with HFrEF and HFpEF. Furthermore, multiple studies have shown that permanent HBP following AVNA significantly improves echocardiographic measurements, NYHA classification, and reduces diuretics use for managing HF in AF patients with narrow QRS who suffer from either HFrEF or HFpEF [37]. Therefore, HPCSP after AVNA is an attractive alternative and pacing mode [43] for patients with both AF and HF with preserved and reduced LVEF, as demonstrated in ongoing clinical trials (NCT02805465, NCT02700425). Evidence from numerous studies supports the short-, and medium-, and long-term efficacy of HPCSP when combined with AVNA in both clinical and echocardiographic outcomes [18, 36, 38].

In addition, combining AVNA with HPCSP has proven effective in improving control over the rate and rhythm of AF with HF. Tong F [34] observed that a high pacing proportion (>71%) through HPCSP significantly improved clinical endpoints in persistent AF patients with either a wide QRS complex or HFrEF, as well those experiencing persistent AF with narrow QRS complex or HFpEF. For these patients, AVNA is recommended to increase the pacing proportion to 100% [34]. Otherwise, combining HPCSP with β-blockers, which reduce the intrinsic heart rate and increase the pacing proportion, has been demonstrated to be an effective therapy for improving both clinical outcomes and echocardiographic parameters in AF patients with HF [34]. Additionally, HPCSP can reverse cardiac remodeling in these patients, as demonstrated by increased ejection fraction (mean difference [MD] = 5.69, 95% confidence interval [CI]: 0.78–10.60, *p* = 0.02), decreased left ventricular end-diastolic diameter (LVEDd) (95% CI: –7.05 to 0.05, *p* = 0.05), and reduced QRS duration (95% CI: –60.71 to –15.88, *p* < 0.01) [44].

It has been shown that HPCSP is superior to BVP in AF patients with HF. Evidence from multiple trials, including the Atrioventricular Junction Ablation and Biventricular Pacing for Atrial Fibrillation and Heart Failure (APAF-CRT) study, demonstrates that the strategy of combining AVNA with CRT significantly reduces all-cause mortality in patients with refractory or highly recurrent AF combined with HF [8]. Unlike CRT, HPCSP is aligned closer to the innate physiological pacing and addresses some limitations of CRT. The first multicenter, prospective, single-blinded, randomized, controlled trial contrasting His-CRT with BVP in patients suitable for CRT enrolled 41 patients [45]. The results demonstrated that patients receiving His-CRT had greater QRS narrowing compared to those with BVP (125 ± 22 ms vs 164 ± 25 ms for total recovery [TR], *p* < 0.001; 124 ± 19 ms vs 162 ± 24 ms for pulse pressure [PP], *p* < 0.001) [45]. Analysis of the echocardiographic response showed a non-significant trend towards an increase (80 vs 57% TR, *p* = 0.14; 91% vs 54% PP, *p* = 0.078) [45]. Another randomized controlled trial led by Vinther *et al*., [46] compared His-CRT with BVP in 50 symptomatic HF patients with HF, LVEF ≤35%, and LBBB. After six months, those in the His-CRT group showed a significantly improved LVEF (48 ± 8% vs. 42 ± 8%; *p* < 0.05) and a reduction in the end-systolic volume (65 ± 22 mL vs. 83 ± 27 mL; *p* < 0.05) when compared to patients treated with BVP [46]. The multicenter randomized controlled The His Bundle Pacing versus Coronary Sinus Pacing for Cardiac Resynchronization Therapy (His-SYNC) study demonstrated that HBP achieved better electrical synchronization and significantly improved cardiac function compared to BVP in patients with NYHA II–IV classification and a QRS duration >120 ms [45]. Moreover, a meta-analysis of 13 studies involving 1121 patients showed that CRT patients with HPCSP led to shorter QRS durations (95% CI: –34.54 to –17.92, *p* < 0.001), increased LVEF (95% CI: 4.81–7.22, *p* < 0.001), lower left ventricular end-diastolic dimension (LVEDD) (95% CI: –4.86 to –0.95, *p =* 0.004), and improved NYHA functional classification (95% CI: –0.67 to –0.23, *p* < 0.001) compared with BVP [19]. Additionally, HPCSP was associated with higher clinical (odds ratio [OR]: 2.10, *p* = 0.01), and optimal clinical (OR: 3.17, *p* < 0.001) responses over BVP, and a decreased hospitalization rate for HF (OR: 0.34, *p* < 0.001) [19]. A comprehensive network meta-analysis of 14 articles involving 2054 patients showed that HBP intervention significantly reduced the QRS duration (MD: –53 ms; 95% CI: –67 to –39) compared with BVP and conventional RVP [47]. The largest network meta-analysis to date, involving 4386 patients, indicated that HPCSP provides advantages over RVP and BVP in patients needing anti-bradycardia pacing and those with HF related to bundle branch block (BBB) [48]. Notably, LBBP resulted in a statistically significant increase in LVEF relative to HBP and BVP (*p* = 0.0001 and *p* = 0.024, respectively), especially in the sensitivity analysis including patients indicated for CRT [48].

## 4. AVNA Combined with HPCSP Performed with Persistent AF Improved the Outcomes

The “pace and ablate” strategy shows potential for improving clinical outcomes for patients with persistent AF [49, 50]. This approach can improve ventricular rate control and adjust the R-R interval [50]. Early studies have confirmed that AVNA combined with ventricular pacing significantly reduces mortality, with a decrease of 55% to 60% [51, 52, 53]. Employing AVNA sequentially with HPCSP is considered a last-resort treatment for a symptomatic persistent AF that is refractory to multiple ablations [54]. This method helps maintain a regular ventricular rhythm, effectively controls the ventricular rate, and reduces or eliminates the need fro antiarrhythmic drugs [54].

Vijayaraman *et al*. [55], compared the clinical outcomes between CP (conventional pacing, either RVP or BVP) and CSP (conduction system pacing, HBP or left bundle-branch area pacing (LBBAP)) in 223 patients undergoing AVNA (CSP, 110; CP, 113). After a mean follow-up of 27 ± 19 months, LVEF in the CSP significantly increased from 46.5% ± 14.2% to 51.9% ± 11.2% (*p* = 0.02), compared to an increase from 36.4% ± 16.1% to 39.5% ± 16% (*p* < 0.04) in the CP group [55]. Additionally, the primary endpoint of time to death or heart failure hospitalization was significantly reduced in the CSP group when compared to the CP group (48% vs 62%; hazard ratio [HR] 0.61, *p* < 0.01) [55]. Further evidence from older patients (≥65 years) with symptomatic persistent AF refractory to repeated ablations, who underwent combined HPCSP and AVNA, demonstrated the significantly reduced symptoms and improved quality of life during a short-term follow-up [21]. In patients with AF, with or without HF, who do not respond to pulmonary vein isolation, AVNA and HPCSP lead to less hemodynamic deterioration and significant improvement in EF [56]. Similarly, Moriña-Vázquez *et al*. [41] demonstrated the effectiveness of AVNA and HBP in patients with poorly controlled AF, achieving strict HR control and full physiological ventricle stimulation. Patients with normal to moderately reduced LVEF, who are unsuitable for CRT, were identified as excellent candidates for permanent HBP after AVNA when both drug and catheter-based therapies fail to control the ventricular rate and symptoms [57]. A recent study found that 13% of patients with “permanent” AF surprisingly regained spontaneous sinus rhythm after being treated with AVNA and HPCSP over a 12-month follow-up [58]. It is hypothesized that this effect occurred through a latent reverse atrial remodeling mechanism [59].

In summary, AVNA produces a slow but stable intrinsic escape rhythm, which allows for effective control over the ventricular rate, achieving full pacing capture of the heart’s rhythm. Concurrently, HPCSP ensures a regular ventricular rhythm. Therefore, the combined rate-and-rhythm strategy effectively manages refractory AF, making it a preferred treatment for persistent AF. Upcoming data from ongoing clinical trials (Resynchronization for Ambulatory Heart Failure Trial in Patients With Chronic Atrial Fibrillation - Pharmacological Rate Control vs. Pace and Ablate With Bi-Ventricular or Conduction System Pacing (RAFT-P&A) trial-NCT06299514) will soon provide further insights.

### Impact of QRS Morphology on Response to“Pace and Ablate” Strategy after AVNA

For patients with AF and a wide QRS duration, undergoing pace and ablate along with an AVNA strategy, both HPCSP and BVP are viable options. However, HBP offers a distinct advantage over BVP and RVP by maintaining ventricular synchronization, even in patients with a normal QRS duration [43]. Specifically, HBP in conjunction with AVNA could provide an additional hemodynamic advantage, potentially outperforming BVP in rate control for refractory AF patients with moderately reduced EF, between 35% and 50%, and narrow QRS [42]. In contrast, BVP may be less suitable and possibly harmful in patients with AF and narrow QRS after AVNA, as it has been associated with increased mortality [60]. Furthermore, studies have shown that the combination of BVP with AVNA is not superior to medication alone in patients with narrow QRS and mid-range LVEF, nor does it improve mortality rates [8]. Therefore, BVP maybe more effective in patients with wide QRS. In patients with a QRS duration of <130 ms, BVP may induce dyssynchrony of ventricular contraction after AVNA [61].

It is possible for HPCSP to reverse ventricle synchrony in patients with both narrow and wide QRS in sinus rhythm [62]. However, patients with LBBB experience greater improvements to LVEF (increasing by 22.3% vs. 14.2%, *p* < 0.001) and NYHA class function (improving from 1.9 to 1.4, *p* < 0.001) compared to those with narrow QRS when undergoing HPCSP with AVNA [63]. Yet nearly 50% of patients with simultaneous HF and LBBB do not achieve QRS normalization through HBP, and the effectiveness of HBP is limited to patients with intraventricular conduction blocks [64]. To address these challenges, the concept of LBBP had been introduced [65]. However, in patients with distal LBB, involvement of the left ventricular Purkinje system, or delayed myocardial conduction that persists despite a wide QRS, pacing the proximal LBB may be ineffective [66]. An optimized CRT (LBB-optimized CRT) combing both LBBP and BVP, has shown promise by achieving a narrower QRS, improving LVEF, and stabilizing pacing parameters [67].

## 5. Difference between LBBP and HBP

While treatment with HBP can provide better physiological ventricular electro-mechanical synchrony than LBBP [44], the technology faces several challenges. These include a prolonged learning curve, poor success rates, longer fluoroscopy duration, higher and unstable pacing thresholds, and early battery depletion [18]. Additionally, only 64% patients successfully undergo HBP implantation [68], and Nearly 5% of those require lead revision due to high thresholds or loss of capture in the course of follow-up [69]. Consequently, the safety concerns associated with high pacing thresholds and low R wave amplitudes limit its use in all pacing applicants, particularly in infra-Hisian block cases [70]. One study suggested that increasing thresholds during AVNA can be mitigated by placing the lead distally in His bundle [18]. Further advancements in tools and technologies are expected to simplify the HBP procedure and increase its success rate.

In terms of interventricular synchrony, LBBP differs from HBP. The unipolar configuration of LBBP generates a mild later contraction of the right ventricle compared to that produced by HBP [71], which mitigates some of HBP’s disadvantages. Therefore, the primary benefits of LBBP include superior pacing characteristics such as lower thresholds, higher sensing amplitudes, and higher success rates, which range from 80% to 97% [72, 73]. More importantly, the risk of increased pacing thresholds involving AVNA is almost non-existent when compared to HBP [74]. Additionally, pacing at the LBB may also inhibit later exacerbation at the proximal His bundle or Atrioventricular node, which may be caused by the progression of AV conduction delay, and may provide additional options (including physical space) for conducting AVNA [37].

Having an adequate distance from the AVNA site, LBBP generates a lower pacing capture threshold and a higher R-wave amplitude compared to HBP, while also stimulating the heart’s conduction system and the deep septal myocardium [14]. Results from a meta-analysis indicate that LBBP achieves a lower pacing threshold (95% CI: 1.12–1.39; *p* < 0.00001) and higher R-wave amplitude (95% CI: –8.46 to –7.31; *p* < 0.00001) when compared to HBP [74]. Another meta-analysis, which included 7 studies with 867 individuals, also indicating that compared with HBP, LBBP is associated with higher procedure success rates (95% CI: 1.05–1.18; *p* = 0.0003), lower capture threshold at both implantation (95% CI: 0.35–0.90, *p* < 0.0001) and during follow-up (95% CI: 0.34–1.18, *p* = 0.0004), and larger sensed R wave amplitudes at both implantation (95% CI: 5.29–9.16, *p* < 0.0001) and during follow-up (95% CI: 6.85–8.22, *p* < 0.0001) [75]. Furthermore, LBBP may enhance physiological pacing and prove more suitable for patients with atrioventricular block (AVB). Li *et al*. [73] investigated LBBP in 33 patients with AVB and found that it had a success rate exceeding 90%, maintained a stable threshold, preserved LV synchronization, and resulted in few complications. In contrast, His bundle pacing enhanced ventricular function and quality of life at the expense of a higher pacing threshold [46].

In studies focusing on lead stability and pacing parameters, LBBP was shown to be superior to HBP. Ye *et al*. [40] demonstrated that LBBP produced better and more stable parameters compared to HBP in the same AF patients, with comparable results observed following the 6-month follow-up. Even in cases where primary HBP is combined with backup LBBP, the advantage lies in the backup LBBP lead’s ability to continue physiological pacing if HBP fails in the future [54]. Combining HBP and LBBP may also be an appropriate strategy as a “pace and ablate” approach for AF that is unresponsive to medical therapy [76].

A recent meta-analysis involving 1035 patients assessed the feasibility, endpoints, and implementation success rates of HBP and LBBP in patients with atrioventricular block (AVB) and preserved left ventricular function [77]. The findings revealed that LBBP resulted in higher R-wave amplitudes (95% CI: 7.26–8.50, *p* < 0.0001) and lower pacing thresholds (95% CI: –0.81 to –0.47, *p* < 0.0001) when compared to HBP [77]. Additionally, LBBP had shorter implantation time (95% CI: –30.44 to –5.18, *p* = 0.006) and reduced fluoroscopy time (95% CI: –8.81 to –1.97, *p* = 0.002).

For pacing-dependent patients, particularly those whose block site is below the His bundle, LBBP is an ideal choice. A prospective multicenter observational study involving 100 patients with HF with reduced EF and LBBB undergoing CRT compared LBBP-CRT (n = 49) with BVP- adaptive CRT (aCRT) (n = 51) [78]. The study demonstrated that LBBP facilitated better resynchronization and elicited higher clinical and echocardiographic responses [78]. A meta-analysis involving six studies with 174 patients indicated that LBBP is both feasible and effective for achieving electric resynchronization and enhancing LV function in CRT candidates [40]. Liu *et al*. [79] demonstrated that LBBP could improve both LV early diastolic function and brain natriuretic peptide (BNP) levels short term in pacemaker-dependent patients compared to right ventricular outflow tract septal pacing. In 2021, Wu *et al*. [80] investigated the long-term feasibility and safety of LBBP, determining that it holds promise to replace HBP and develop into a more widely applicable physiological pacing strategy.

Recent studies have explored the efficacy of different pacing sites in patients with complete AVB undergoing pacemaker implantation. Chen *et al*. [81] conducted a study on 20 patients with complete AVB who underwent dual-chamber pacemaker implantation. They found that LBBP produced a paced QRS duration (QRSd) of 116.15 ± 11.60 ms, which was narrower than that achieved with other pacing techniques [81]. Specifically, QRSd for LBBP was narrower compared to the ring electrode of the LBBP lead (RVSP ring) at 135.11 ± 13.68 ms, right ventricular septum pacing (RVSP) at 141.65 ± 14.26 ms, and right ventricular apex pacing (RVAP) at 160.15 ± 19.35 ms (*p* < 0.001) [81]. This performance was comparable to His bundle pacing (HBP), which showed a QRSd of 114.84 ± 18.67 ms [81]. Additionally, success rates and reductions in QRS duration were similar between LBBP and HBP [77].

Recent meta-analyses have explored the comparative outcomes of different physiological pacing patterns. One such analysis revealed no significant statistical difference in the incidence of outcomes between two physiologic pacing patterns (RR = 1.56, 95% CI: 0.87–2.80, *p* = 0.14) [8]. Additionally, the study also found that LBBP may enhance the QRS duration and carries potential risks such as right bundle branch lesions and septal perforation [8]. Furthermore, LBBP can produce right ventricular activation delay, indicating it may be inferior to HBP [8]. Despite these concerns, LBBP is still considered a viable alternative for patients with pacing dependence [82], although its long-term safety remains still uncertain. Reflecting this, the recent Heart Rhythm Society (HRS) guidelines have categorized left bundle branch pacing as a Class IIb recommendation [83].

## 6. Disadvantages and Future of HPCSP

Although HPCSP maintains excellent electrical activation and mechanical contraction synchronization in the ventricles, it faces specific limitations, particularly with HBP. Owing to the anatomical characteristics of the His bundle, the detectability of the His bundle during pacing is low, leading to increased pacing thresholds after follow-up. A meta-analysis [13] confirmed that HBP is associated with a rise in the pacing threshold, which could shorten the life of the pacemaker in patients reliant on long-term pacing. Therefore, the pacemaker replacement rate after 5 years of HBP is significantly higher compared to right ventricular pacing [11]. In contrast, LBBP maintains a low and stable threshold, circumventing many disadvantages associated with HBP [84, 85]. Implementing LBBP as an alternative if HBP fails or proves inadequate may be a promising strategy [21].

Other challenges with HPCSP include the difficulty of achieving precise localization and stable fixation of the lead, with current implantation tools often inadequate for the task. This typically results in prolonged implantation and fluoroscopic times, as well as increased battery depletion. However, the advent of three-dimensional mapping system may facilitate and simplify the selection of implantation sites for HBP [86]. To conclusively assess the efficacy and safety of AVNA with HBP compared to drug therapy, AVNA with BVP, and catheter ablation of AF, large, prospective, and randomized controlled trials are essential.

## 7. Conclusions

The strategy of AVNA combined with HPCSP could significantly improve LV function, demonstrating robust short to medium term efficacy and safety in AF patients with HF. This approach holds a significant advantage over BVP and RVP in maintaining ventricular systolic synchronization for patients with a normal QRS duration. Although HBP may be challenging due to difficulties in precise localization and increased pacing thresholds, LBBP offers lower thresholds, higher sensing amplitudes, and greater success rates, potentially replacing HBP. We are fortunate to be in an era where multiple pacing strategies are available for patients with HF. Larger, prospective, and randomized controlled studies are needed to evaluate patient selection and implantation technology, aiming to identify differential clinical endpoints between pacing modalities.
